# Supplementary material for: ATP plays a structural role in Hsp90 function
Source: Nat Commun. 2025 Jul 21;16:6710. doi: 10.1038/s41467-025-61962-0 (PMC12280139; doi:10.1038/s41467-025-61962-0)
Supplement: Supplementary file 1 — Supplementary Information [file 41467_2025_61962_MOESM1_ESM.pdf]

Supplemental Information

Supplemental Figure 1

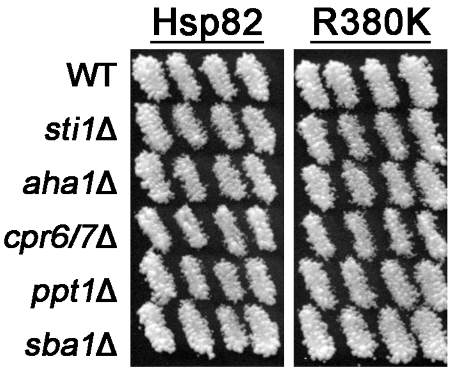

Supplemental Figure 1. Hsp82^{R380K} is not dependent on Hsp90 co-chaperones for *in vivo* function. *In vivo* Hsp90 functional assay with wild type Hsp90 (left column) or Hsp82^{R380K} (right column) expressed in yeast strains lacking the indicated Hsp90 co-chaperones. Images were cropped from the same plate.

Supplemental Figure 2

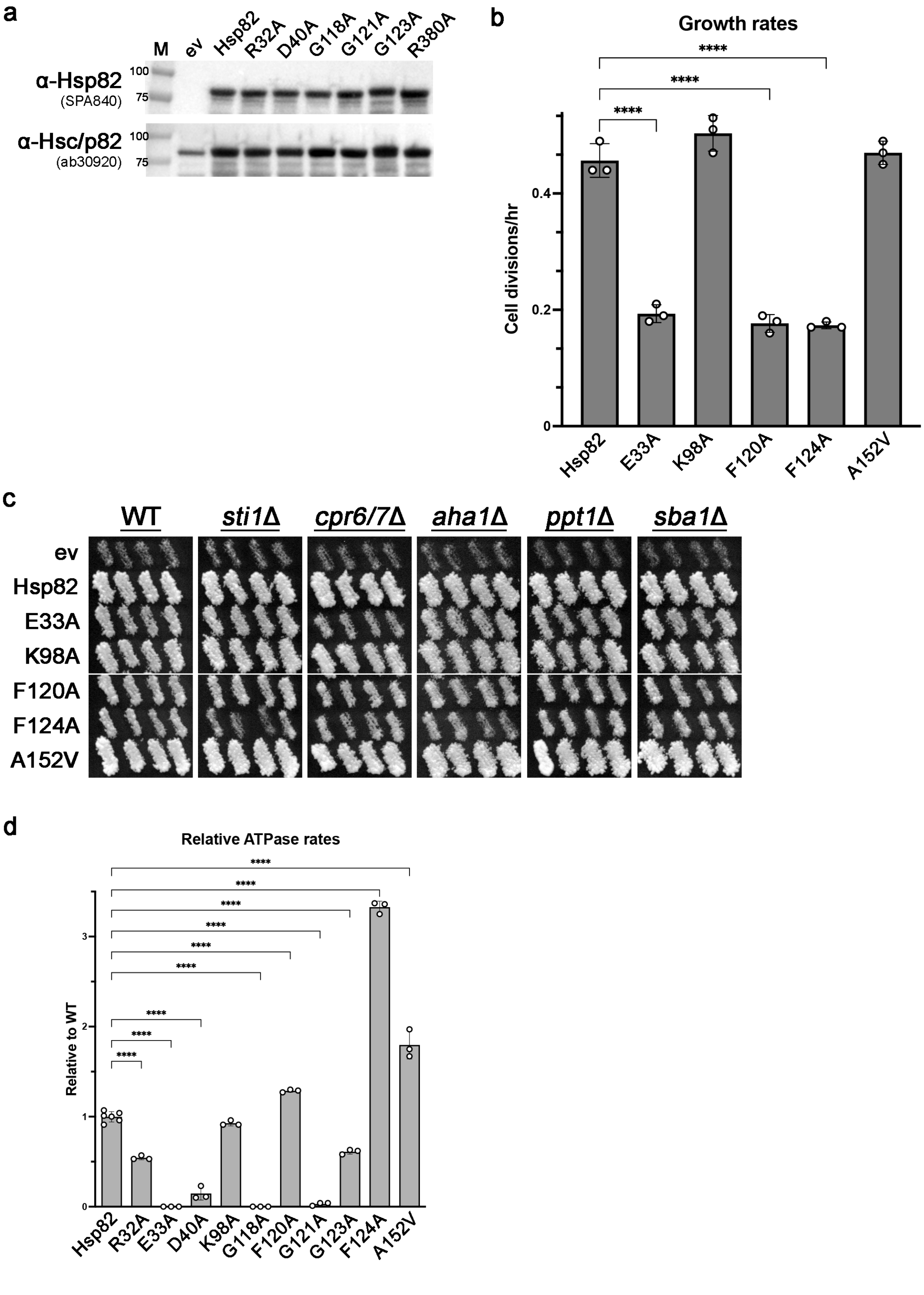

Supplemental Figure 2. a. Western blot showing the expression of the mutant Hsp82 proteins in cells expressing wild type Hsc82 to maintain viability, as described in Methods. Antibody SPA-840 (top) recognizes only Hsp82, while anti-Hsc/p82 (bottom) recognizes both yeast Hsp90 isoforms. **b.** *In vivo* Hsp90 functional assay with the indicated Hsp82 mutants on left expressed in the co-chaperone deletion strains as indicated at top. All images are cropped from three different plates of identical composition. Note, the “WT” panel is reproduced from the left panel in Figure 6b. **c.** Growth rates of cells expressing the indicated Hsp82 mutants as the sole source of Hsp90 were measured as described in Methods. Shown are the inverse doubling times; bars are the average of three independent biological replicates and the error bars are the standard deviation. Asterisks indicate significant differences compared to wild type ($p < 0.0001$ by one-way ANOVA). **d.** ATPase rates of the indicated Hsp82 mutant proteins relative to wild type Hsp82. Asterisks indicate significant difference from wild type ($p < 0.0001$ by one-way ANOVA).

Supplemental Figure 3

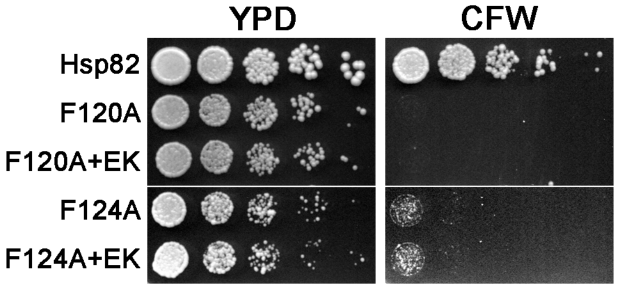

Supplemental Figure 3. E372K does not rescue the F120A or F124A growth defects. Cells expressing the indicated Hsp82 mutant were spotted onto YPD and CFW as described in Methods. Lines in the images indicate where portions were cropped from different plates of identical composition.

Supplemental Table 1. Primers used in this study.

**Name**	**Sequence (5’ to 3’)**
Cdc37-500-HI-F	GATCggatccATGCCTACGAATATTCCAGG
Cdc37+500-HI-R	GATCggatccAGAATTCTTTACCAGTTCCA
Cdc37-in-pMR520-GA-F	gatccccgggtaccgagctcATGCCTACGAATATTCCAGG
Cdc37-in-pMR520-GA-R	cggccagtgaattcgagctcAGAATTCTTTACCAGTTCCA
Sti1-Nhe1-Fw	gtcagcGCTAGCATGTCATTGACAGCCGATG
Sti1-Xho1-Rev	gtcagtCTCGAGTTAGCGGCCAGTCCGGATG
Hsp82-R32A-F	CTAACAAGGAAATTTTCTTGGCTGAACTGATATCTAATGCCTC
Hsp82-D40A-F	ACTGATATCTAATGCCTCGGCTGCGTTGGATAAAATTAGAT
Hsp82-K98A-F	ATAACTTGGGTACCATTGCCGCTTCTGGTACCAAAGCCTTCAT
Hsp82-G118A-F	TGCCGATGTATCCATGATTGCTCAATTCGGTGTTGGTTTTT
Hsp82-F120A-F	ATGTATCCATGATTGGTCAAGCTGGTGTTGGTTTTTACTCTTT
Hsp82-F120C-F	ATGTATCCATGATTGGTCAAtgtGGTGTTGGTTTTTACTCTTT
Hsp82-F120D-F	ATGTATCCATGATTGGTCAAgatGGTGTTGGTTTTTACTCTTT
Hsp82-F120E-F	ATGTATCCATGATTGGTCAAGAAGGTGTTGGTTTTTACTCTTT

Hsp82-F120G-F	ATGTATCCATGATTGGTCAAggtGGTGTTGGTTTTTACTCTTT
Hsp82-F120H-F	ATGTATCCATGATTGGTCAACATGGTGTTGGTTTTTACTCTTT
Hsp82-F120I-F	ATGTATCCATGATTGGTCAAattGGTGTTGGTTTTTACTCTTT
Hsp82-F120K-F	ATGTATCCATGATTGGTCAAAAAGGTGTTGGTTTTTACTCTTT
Hsp82-F120L-F	GTATCCATGATTGGTCAATTGGGTGTTGGTTTTTACTCTTT
Hsp82-F120M-F	ATGTATCCATGATTGGTCAAatgGGTGTTGGTTTTTACTCTTT
Hsp82-F120N-F	ATGTATCCATGATTGGTCAAaatGGTGTTGGTTTTTACTCTTT
Hsp82-F120P-F	ATGTATCCATGATTGGTCAAccaGGTGTTGGTTTTTACTCTTT
Hsp82-F120Q-F	ATGTATCCATGATTGGTCAACAAGGTGTTGGTTTTTACTCTTT
Hsp82-F120R-F	ATGTATCCATGATTGGTCAAagaGGTGTTGGTTTTTACTCTTT
Hsp82-F120S-F	TGTATCCATGATTGGTCAATCTGGTGTTGGTTTTTACTCTTT
Hsp82-F120T-F	ATGTATCCATGATTGGTCAAactGGTGTTGGTTTTTACTCTTT
Hsp82-F120V-F	ATGTATCCATGATTGGTCAAgttGGTGTTGGTTTTTACTCTTT
Hsp82-F120W-F	ATGTATCCATGATTGGTCAAtggGGTGTTGGTTTTTACTCTTT
Hsp82-F120Y-F	TGTATCCATGATTGGTCAAtatGGTGTTGGTTTTTACTCTTT
Hsp82-G121A-F	ATCCATGATTGGTCAATTCGCTGTTGGTTTTTACTCTTTAT
Hsp82-G123A-F	GATTGGTCAATTCGGTGTTGCTTTTTACTCTTTATTCTTAG
Hsp82-F124A-F	TTGGTCAATTCGGTGTTGGTGCTTACTCTTTATTCTTAGTTG
Hsp82-A152V-F	ATACATCTGGGAATCCAACGTTGGTGGTTCTTTCACTGTTA
P82-E372K-F	TCAAGGGTGTTGTTGACTCTAAAGATTTACCATTGAATTTGTC
Hsp82-R380A-F	ATTTACCATTGAATTTGTCCGCCGAAATGTTACAACAAAATAA
Hsp82-R380K-F	TTTACCATTGAATTTGTCCAAGGAAATGTTACAACAAAATAA
