# Supplementary material for: ATP plays a structural role in Hsp90 function
Source: Nat Commun. 2025 Jul 21;16:6710. doi: 10.1038/s41467-025-61962-0 (PMC12280139; doi:10.1038/s41467-025-61962-0)

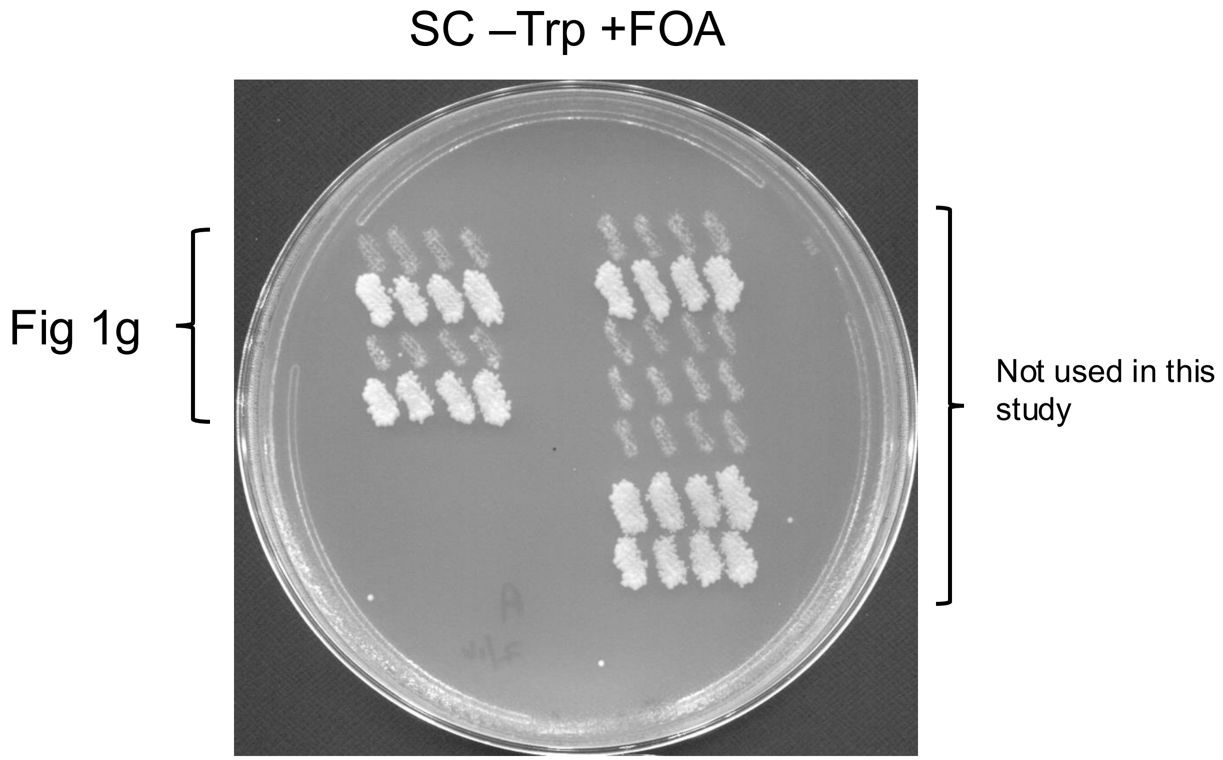
SC –Trp +FOA
Fig 1g
Not used in this study

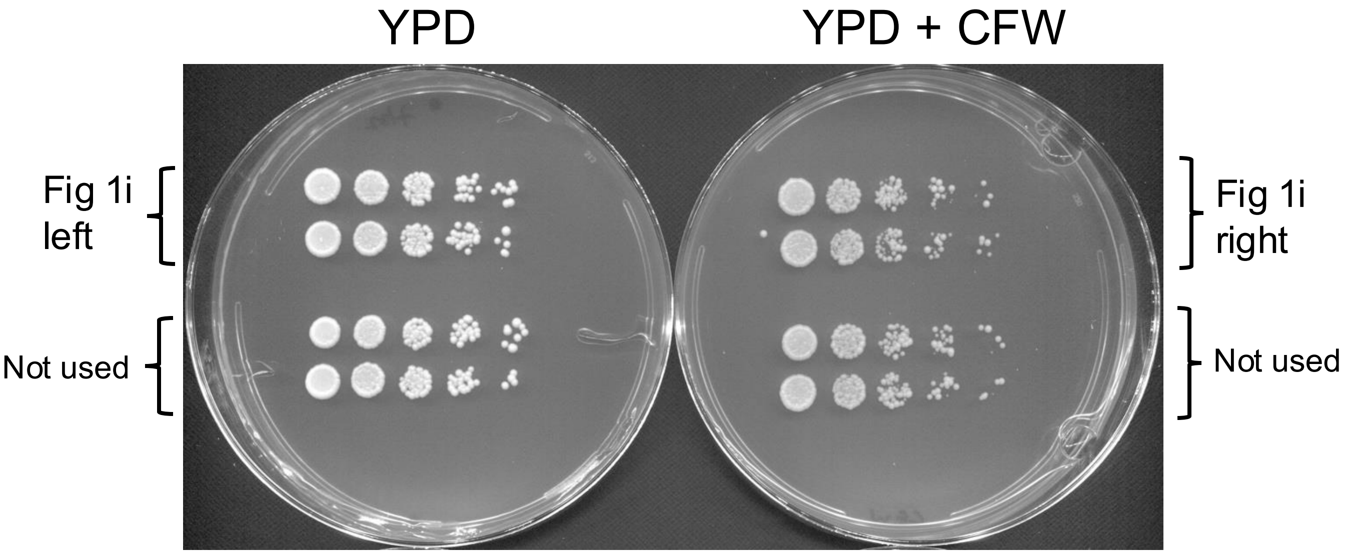
YPD
YPD + CFW
Fig 1i left
Not used
Fig 1i right
Not used

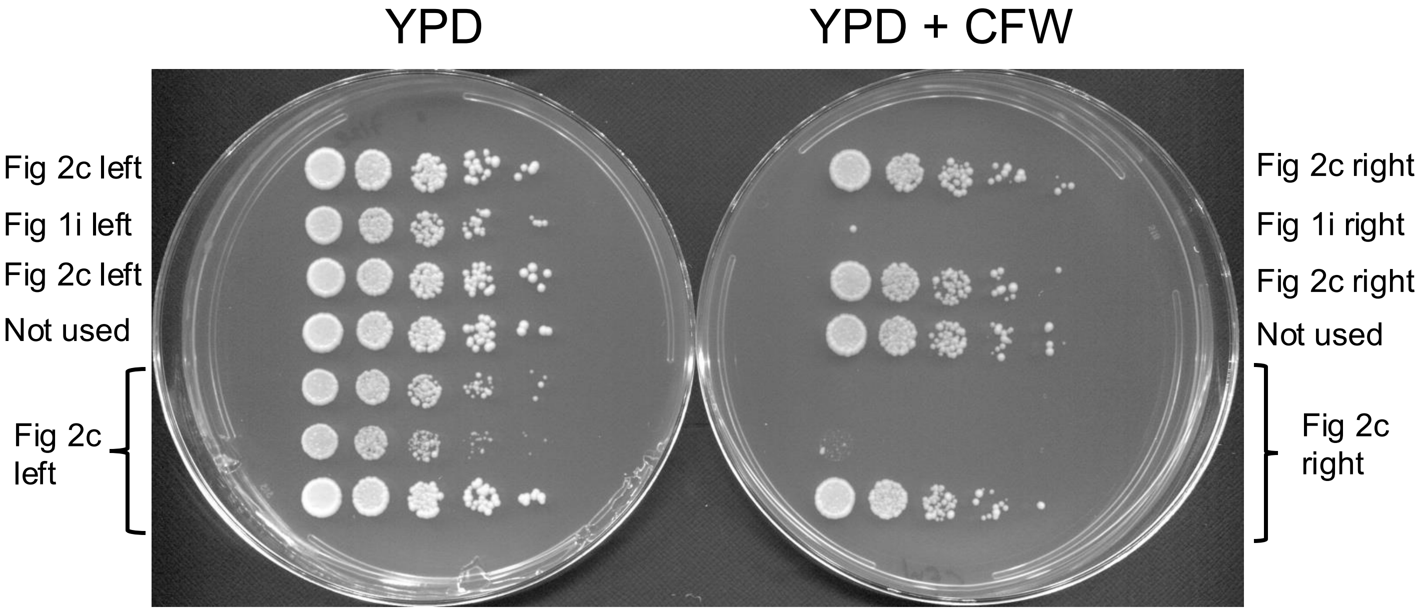
YPD
YPD + CFW
Fig 2c left
Fig 1i left
Fig 2c left
Not used
Fig 2c left
Fig 2c right
Fig 1i right
Fig 2c right
Not used
Fig 2c right

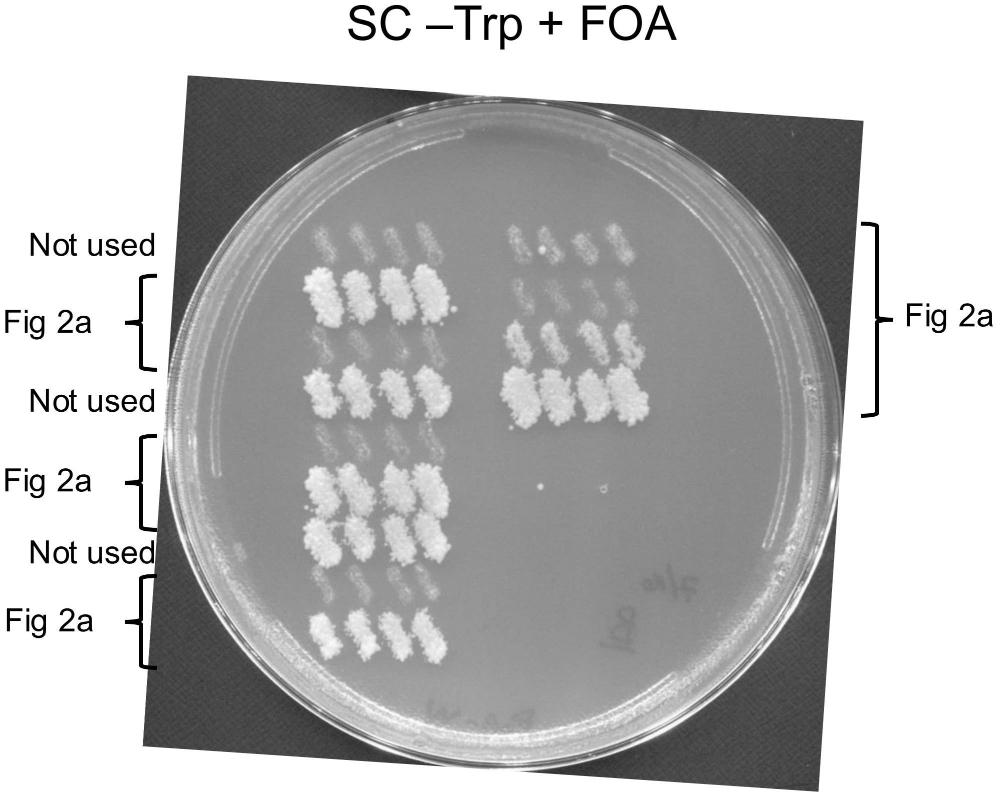
SC –Trp + FOA
Not used
Fig 2a
Not used
Fig 2a
Not used
Fig 2a
Fig 2a

SC –Trp + FOA

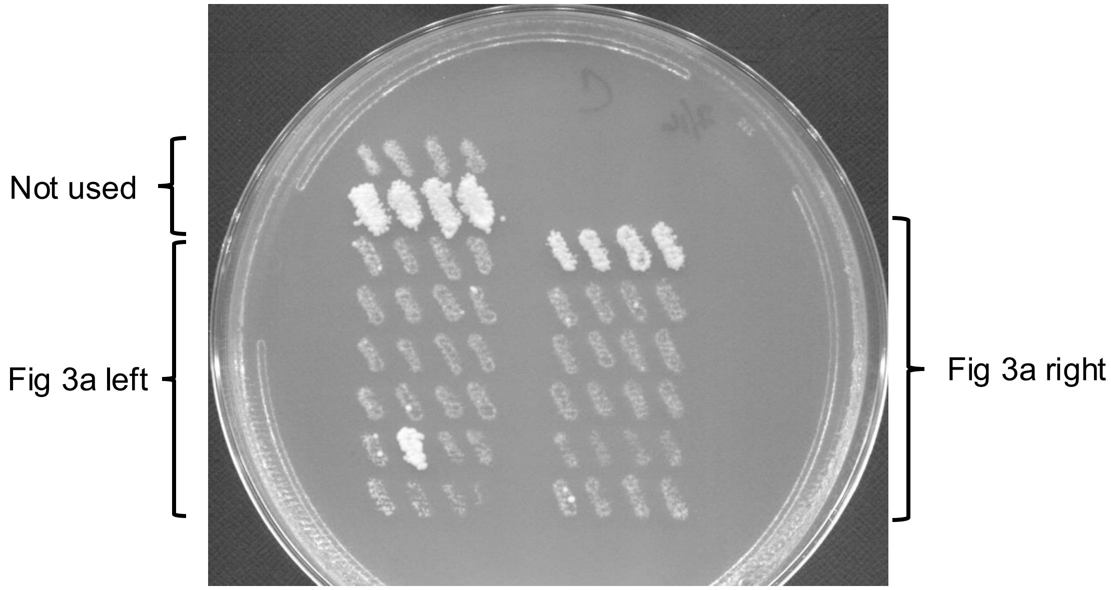
Not used
Fig 3a left
Fig 3a right

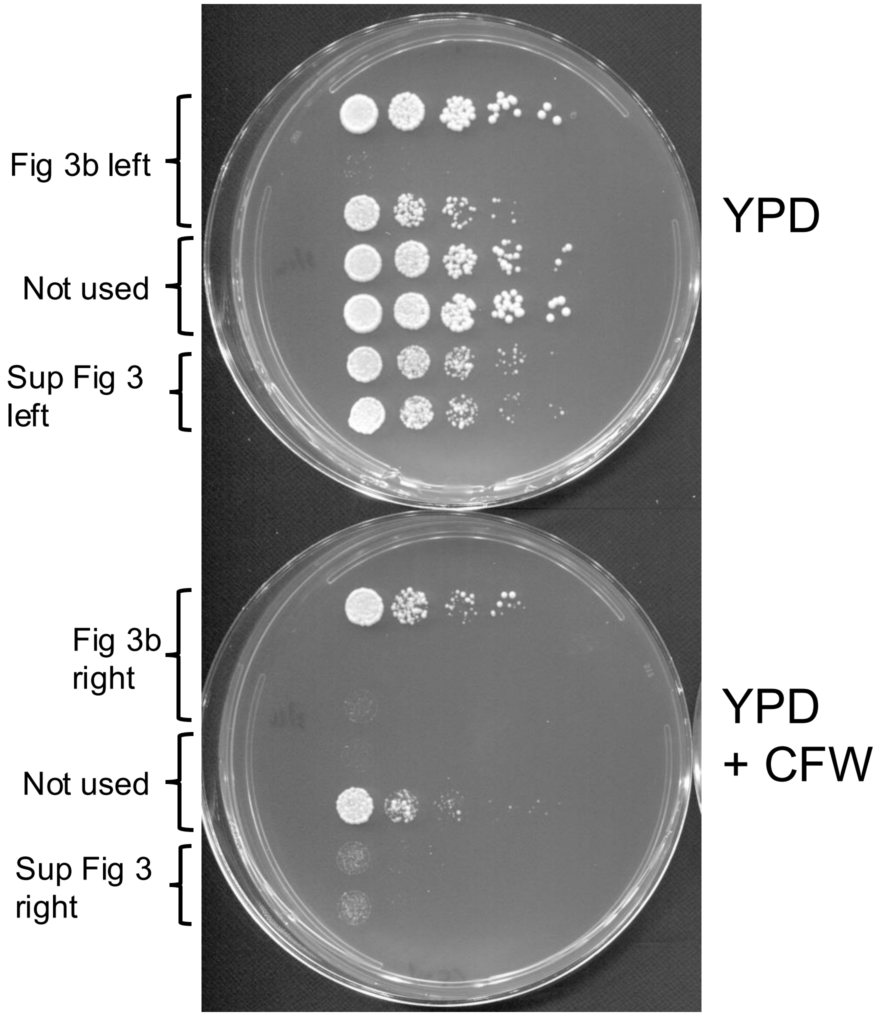
Fig 3b left
Not used
Sup Fig 3 left
YPD
Fig 3b right
Not used
Sup Fig 3 right
YPD + CFW

SC –Trp + FOA

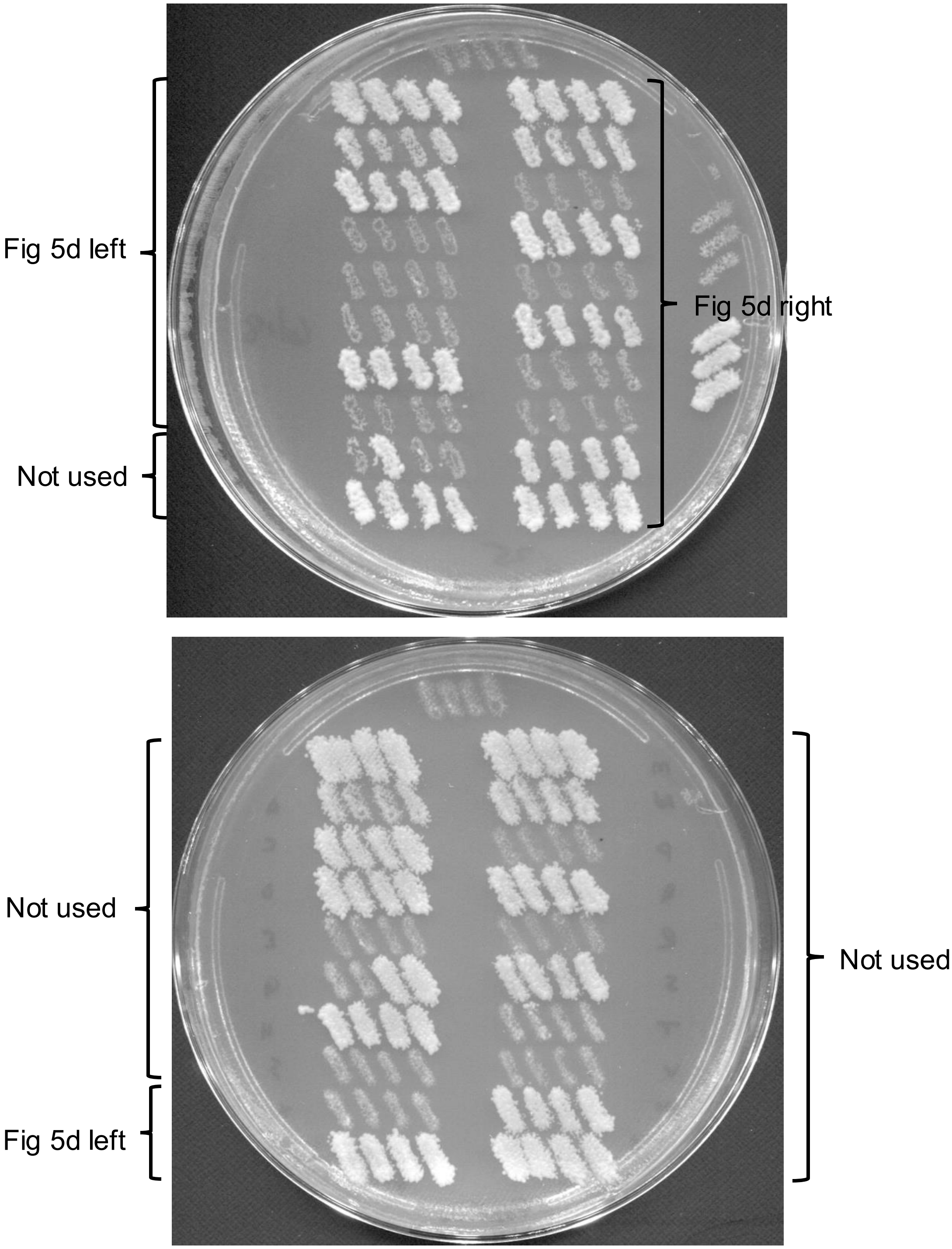

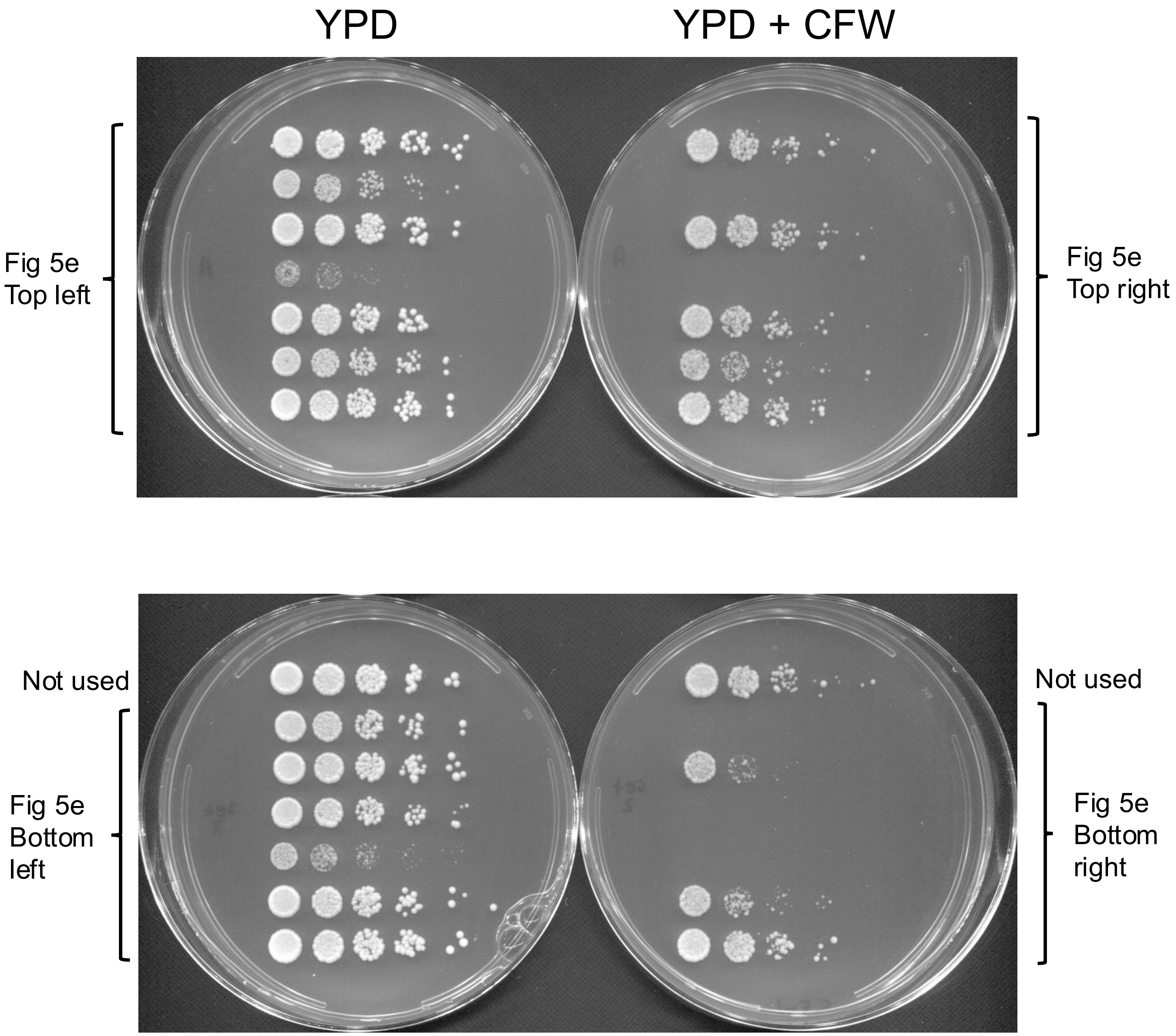
YPD
YPD + CFW
Fig 5e
Top left
Fig 5e
Top right
Not used
Not used
Fig 5e
Bottom
left
Fig 5e
Bottom
right

SC –Trp –His + FOA

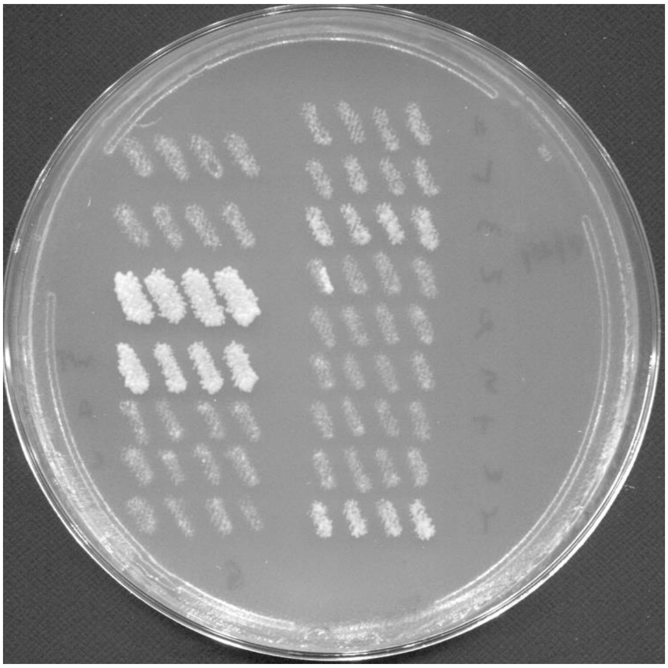

SC –Trp + FOA

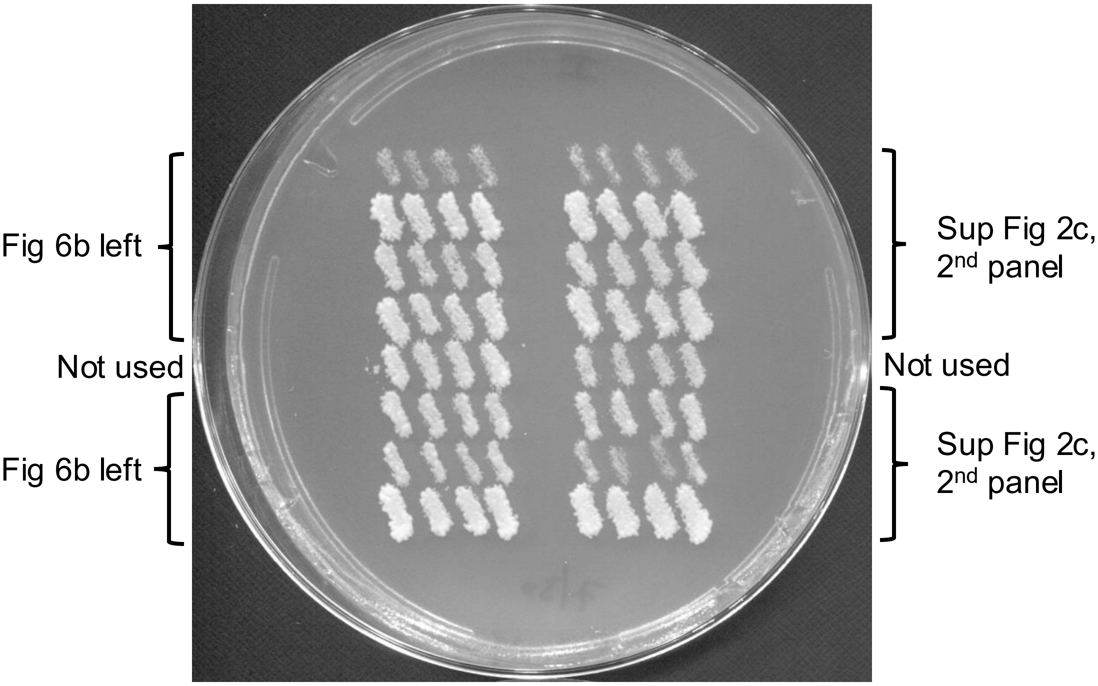

SC –Trp –His + FOA

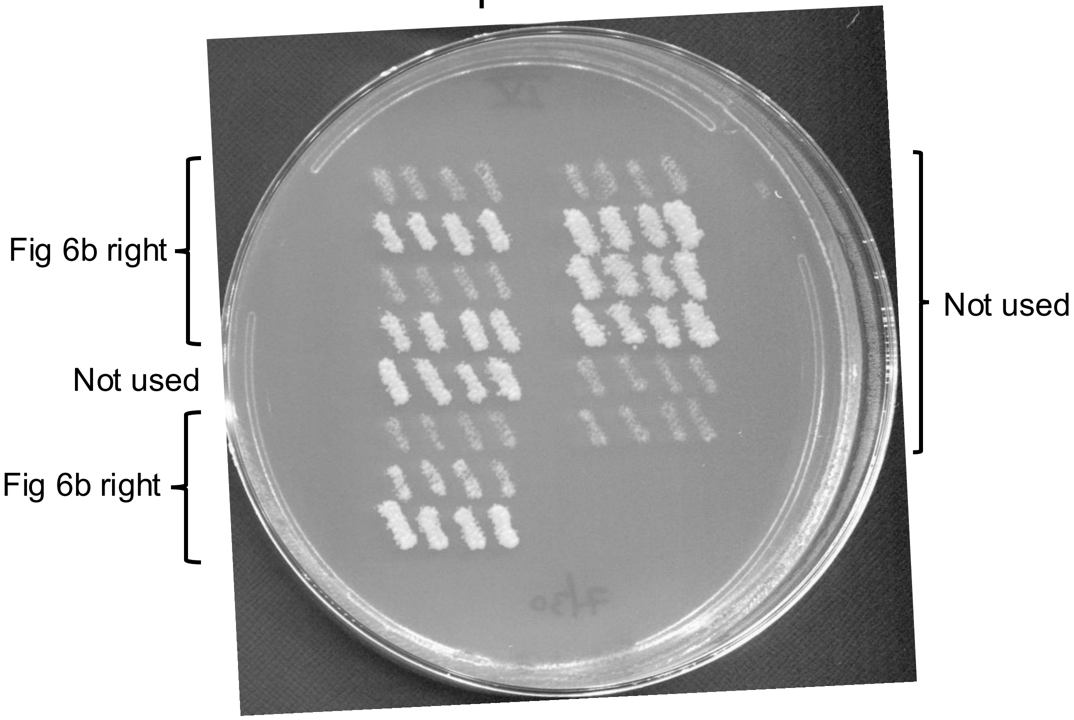

SC –Trp + FOA

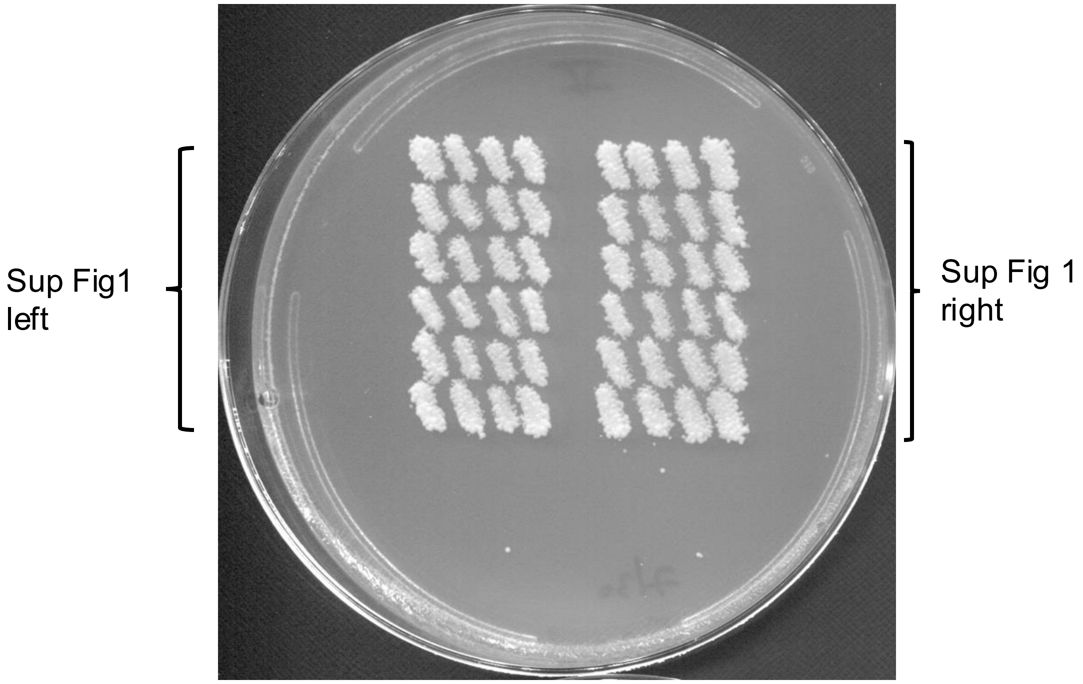

SC –Trp + FOA

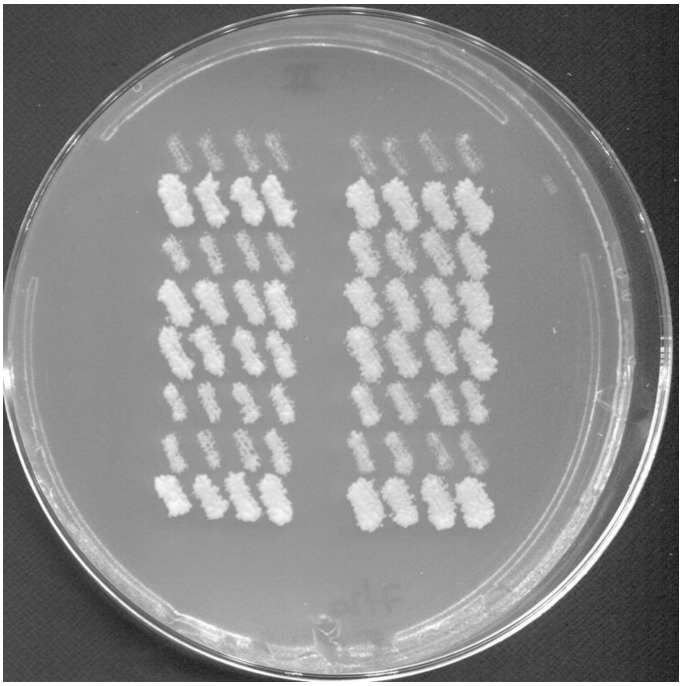

Sup Fig 2c 3rd panel

Not used

Sup Fig 2c 3rd panel

Sup Fig 2c, 4th panel

Not used

Sup Fig 2c, 4th panel

SC –Trp + FOA

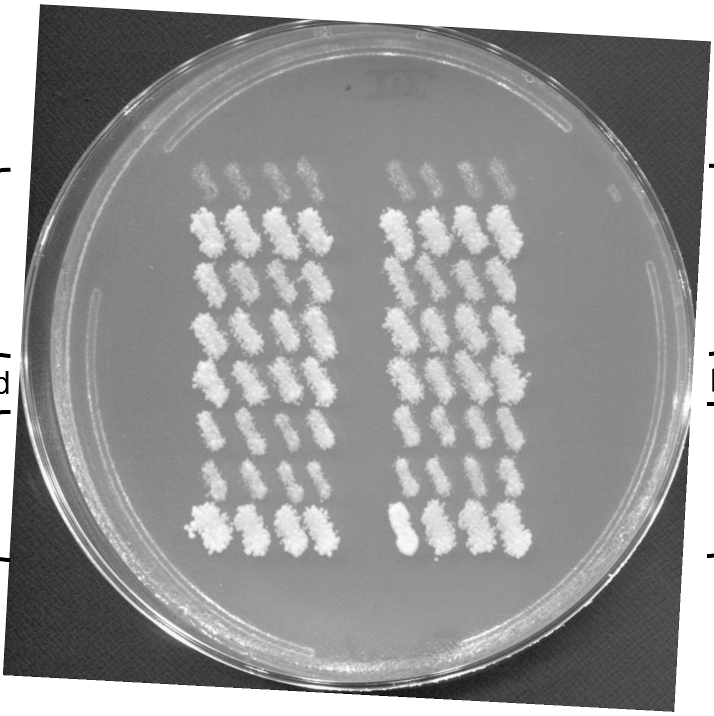

Sup Fig 2c 6th panel

Not used

Sup Fig 2c 6th panel

Sup Fig 2c, 5th panel

Not used

Sup Fig 2c, 5th panel

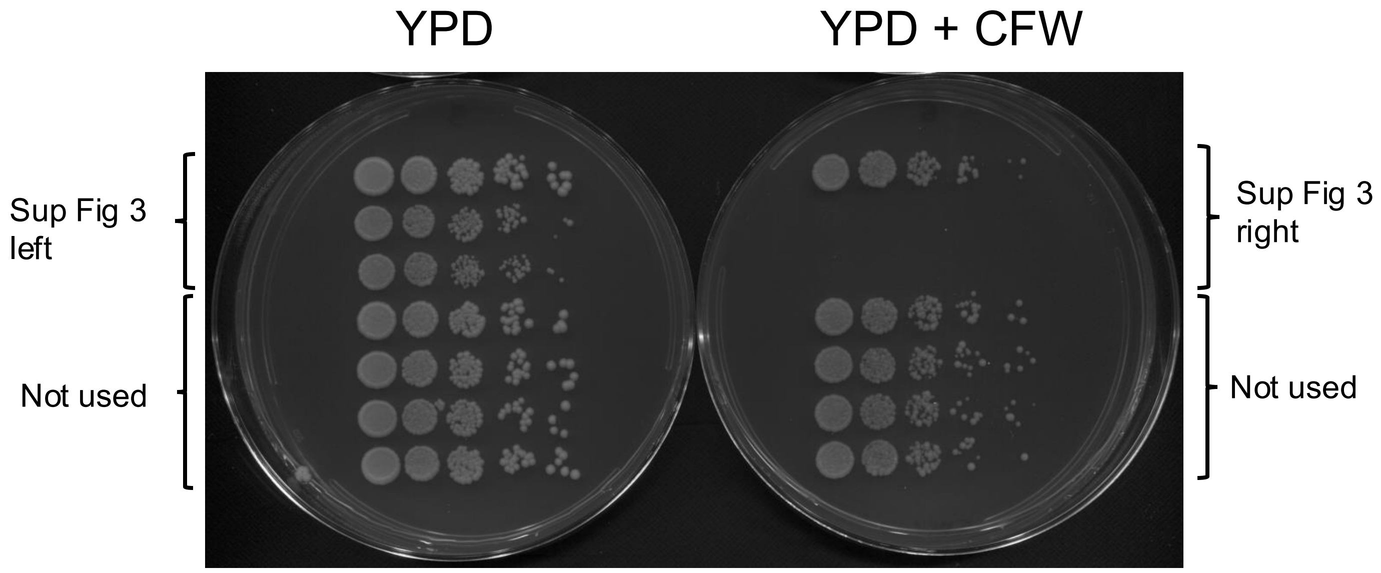
YPD
YPD + CFW
Sup Fig 3 left
Not used
Sup Fig 3 right
Not used

Sup Fig 1a uncropped blots

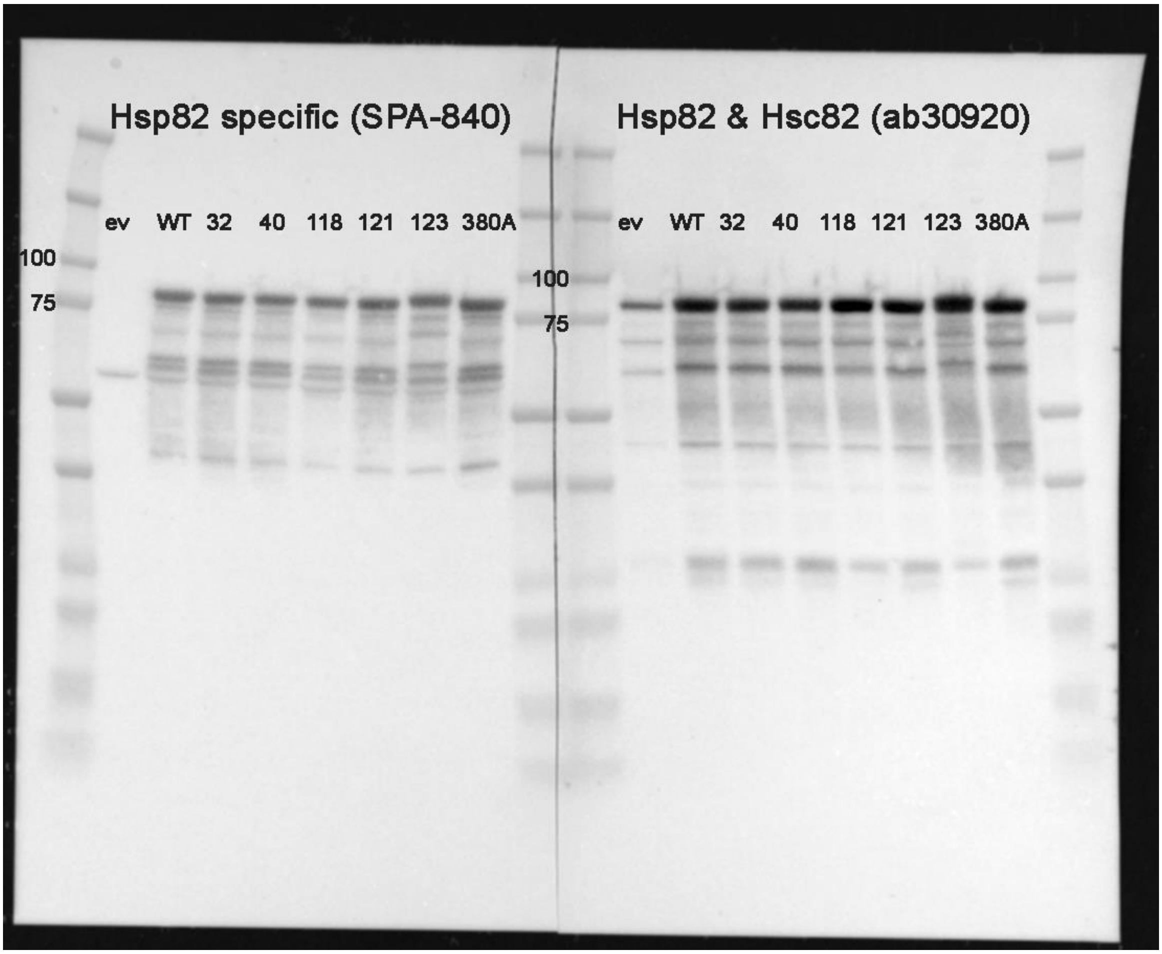

Loading control (stained membranes)

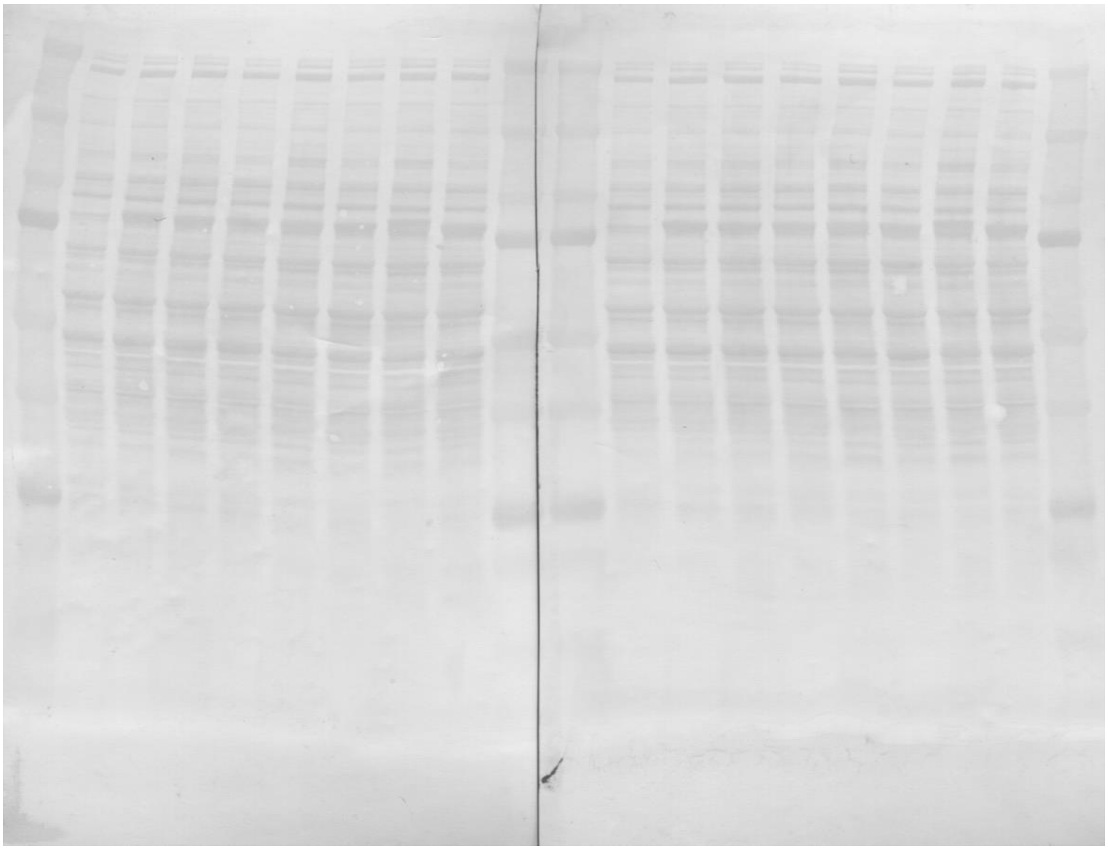

Supplement: Supplementary file 4 — Source Data [file 41467_2025_61962_MOESM4_ESM.zip › uncropped plates and blots.pdf]
